# Dramatic early event in chronic allograft nephropathy: increased but not decreased expression of MMP-9 gene

**DOI:** 10.1186/1746-1596-8-13

**Published:** 2013-01-28

**Authors:** Dongfeng Gu, Yanling Shi, Yanan Ding, Xinyu Liu, Hequn Zou

**Affiliations:** 1 Department of Nephrology, The Third Affiliated Hospital of Southern Medical University, Guangzhou, 510630, People’s Republic of China; 2 Department of Nephrology, Wenzhou Medical College, Wenzhou, 325035, People’s Republic of China; 3 Department of Cardiovascular Medicine, The Third Affiliated Hospital of Southern Medical University, Guangzhou, 510515, People’s Republic of China

**Keywords:** Chronic allograft nephropathy (CAN), Mononuclear cells, Matrix metalloproteinase 9 (MMP-9), Transforming growth factor-beta1 (TGF-beta1), Smooth muscle cells (SMCs)

## Abstract

**Objective:**

The infiltration of mononuclear cells and replication and migration of smooth muscle cells (SMCs) from media into the intima in the vascular wall are the cardinal pathological changes in the early stage of chronic allograft nephropathy (CAN). But the mechanism is unclear. Therefore we investigated the role of matrix metalloproteinase 9 (MMP-9) and its interaction with TGF-beta1, tubulointerstitial mononuclear cells infiltration and migration of SMCs in the early stage of CAN.

**Methods:**

Kidneys of Fisher (F334) rats were orthotopically transplanted into bilaterally nephrectomized Lewis (LEW) recipients. To suppress an initial episode of acute rejection, rats were briefly treated with cyclosporine A (1.5 mg/kg/day) for the first 10 days. Animals were harvested at 12 weeks after transplantation for histological, immunohistochemistry and molecular biological analysis.

**Results:**

The expression of MMP-9 was up-regulated in interstitium and vascular wall in the early stage of CAN, where there were interstitial mononuclear cells infiltration and SMCs migration and proliferation. Moreover the expression of MMP-9 were positively correlated with the degree of interstitial mononuclear cells infiltration, the quantity of SMCs in arteriolar wall, and also the increased TFG-beta1 expression in the tubulointerstitium and arteriolar wall.

**Conclusions:**

MMP-9 may play an important role in the mechanism of pathological changes during the earlier period of CAN.

**Virtual Slides:**

The virtual slide(s) for this article can be found here: http://www.diagnosticpathology.diagnomx.eu/vs/1582313332832700.

## Introduction

Nowadays, it is well known that long-term renal allograft survival is limited mainly by the progressive process termed chronic allograft nephropathy (CAN) or chronic rejection. Pathological features of CAN consist of diffuse interstitial lymphocytes’ inflammation and fibrosis, glomerular mesangial matrix increase, glomerular sclerosis, vascular intimal proliferation and tubular atrophy. The infiltration of mononuclear cells and the migration of smooth muscle cells (SMCs) from media into the intima of the vascular wall are considered to be the cardinal pathological changes during the early stage of CAN [1-4]. However, the mechanism is unclear. Thus, it is of great significance to understand the underlying mechanism.

In other inflammatory diseases the remodeling of extracellular matrix is a prerequisite for the migration of mononuclear and other cells into the tissue. Gelatinase B (matrix metalloproteinase-9, MMP-9) is a secreted multidomain enzyme that is important for the remodeling of the extracellular matrix and the migration of normal and tumor cells. It cleaves denatured collagens (gelatins) and type IV collagen, which is present in basement membranes. In the immune system, this cleavage helps lymphocytes and other leukocytes to enter and move within the tissue [5].

MMP-9 belongs to the subfamily of MMPs that play an important role in tissue remodeling in normal and pathological inflammatory processes. MMP-9 is a major secretion product of macrophages and a component of cytoplasmic granules of neutrophils [6], and is also secreted by lymphocytes and fibroblasts [7] and vascular SMC [8] upon stimulation by inflammatory cytokines, such as TGF-beta1, IL-1, TNF-α, MCP-1 and RANTES, etc. Our previous study had shown the role of RANTES in CAN [9].

Recently, it has been demonstrated that MMP-9 plays an important role in the migration of mononuclear cells in the tissue of some chronic inflammatory diseases [10-12]. MMP-9 can cleave basement membranes of blood vessel and extracellular matrix. The increase of MMP-9 and cleaved extracellular matrix segments parallels the infiltration of mononuclear cells [5]. MMP-9 produced by monocytes and macrophages and plays an important role in the inflammation [5]. In an experimentally induced animal model of delayed-type hypersensitivity (DTH), injection of MMP-9 lead to the infiltration of leukocytes [13].

Newby et al. provided direct evidence for the participation of MMP-9 in proliferation and migration of SMCs (the process of arteriosclerosis) to the intima in an in vitro experiment of rabbit’s aorta [14]. Aoyagi et al. discovered that MMP-9 played an important role in the migration of SMCs to the intima of the rabbit carotid arteries after balloon denudation [15].

According to the close relations between MMP-9 and the migration of mononuclear cells and SMC, it is very likely that MMP-9 is also an important factor in the earlier stage of CAN.

Thus, we investigated the expression and its role of MMP-9 in the early stage of CAN in a standardized rat model.

## Methods

### Animals

Animal research was approved by the Ethics Committee of the Third Affiliated Hospital of Southern Medical University. Naive inbred male rats weighing 200-250 g (Experimental animal center of China, Peking) were used throughout the experiments. Lewis (LEW) rats acted as graft recipients and Fisher (F334) rats as donors. They were housed under standard conditions at controlled temperature, humidity and light/dark cycles, fed a standard diet and had free access to tap water.

### Drugs

Cyclosporine A (Novartis Pharma AG, Basel, Switzerland) was dissolved in cremophor and administered subcutaneously.

### Surgery

Operative procedures were performed under general anesthesia induced by ketamine (100 mg/kg body weight; Ketamin 2%, Huadong-Pharma, Shanghai, China) administered intraperitoneally. The left donor kidney was isolated, removed, cooled and positioned orthotopically into the host whose left renal vessels had been isolated, clamped, and the native kidney removed. Donor and recipient renal artery, vein, and ureter were then anastomosed end-to-end with 10–0 prolene sutures. No ureteral stent was used. Mean ischemia time was 25 minutes (ranges 22–31min). The remaining right kidney of the recipient was removed on day 10, at which time the transplanted kidney was checked for surgical damage. Rats with any overt signs of unsuccessful operation were discarded.

### Experimental design

Ten male LEW rats received left renal transplantation orthotopically from ten male F334 donors. To suppress acute rejection, rats were treated with low dose CsA (1.5 mg/kg/day) for the first 10 days. Ten uninephrectomized male F334 rats and ten uninephrectomized male LEW rats were used as control animals.

### Functional measurements

At week 12, rats were put into metabolic cages and 24 hour urine samples were collected and quantitative proteinuria was determined. At the same time, serum and urine creatinine were measured and creatinine clearance was calculated.

### Harvesting

At week 12, recipients and controls were sacrificed and the transplanted kidney removed. Only kidneys without apparent complications of grafting such as pyelonephritis or hydronephrosis were evaluated. Representative portions of the kidneys were snap-frozen in liquid nitrogen and stored at −80°C for PCR analysis or fixed in formalin (4%) for histological and immunohistological evaluation.

### Histology

Formalin-fixed and paraffin embedded sections were stained with hematoxylin/eosin to assess the grade of cellular infiltration and tubular atrophy. Periodic acid-Schiff (PAS) reaction was used to evaluate the extent of glomerulo- and arterio-sclerosis. Tissue sections were coded and examined in a blinded fashion by light microscopy. At least 200 glomeruli were counted in each section. Renal structural damage was scored semiquantitatively on a scale from 0 to 3+ for interstitial cellular infiltration, tubulopathy, glomerulopathy and arterial intimal fibroplasia using the Banff criteria [16] and the sum of scores (0–12+) was calculated for each sample.

### Immunohistology

Goat anti-rat polyclonal antibodies MMP-9 and TGF-beta1 are purchased from Santa Cruz (U.S.A.) and for secondary and tertiary staining from DAKO (Denmark).

The antigen on the formalin-fixed and paraffin embedded sections (2 μm) was restored by microwave, and incubated with primary antibodies as mentioned above using the LSAB techniques. The intensity of tissue staining for MMP-9 and TGF-beta1 was evaluated in a blinded manner by calculating the relative stained area using a computerized pathological image analytical system.

Images were acquired by means of Leica DMR-X microscope coupled to a Leica DC500 digital camera (Leica, Wetzlar, Germany) and the image analysis system Quantimet Q550 (Leica Imaging Systems). Ten randomly selected discontinuous fields (400 ×) per kidney were evaluated, including tubulointerstitial in renal cortex, medulla and the conjunction region. More than 60 tubules in each biopsy section were observed. The positive area was yellow staining and Image-Pro Plus software was used to quantify the integrated optical density. There were four classed intensity of staining (negative vs. pale yellow vs. pale brown vs. russet) and four classed staining area (staining area < 25% vs. staining area 25–50% vs. staining area 50–75% vs. staining area > 75%). product of intensity and relevant staining area were used for statistical analysis.

### RNA isolation

Total RNA was extracted from the representative portions of the kidneys using Trizol (Gibco/BRL, U.S.A.), which is based on the method described by Chomczynski and Sacchi [17].

Representative portions of the kidneys samples were stored in −80°C. 60–100 mg frozen tissue samples were homogenized in 1ml Trizol. 200 μl chloroform was added to each mixture. The mixture was centrifuged at 12,000 g for 15 min at 4°C. The supernatant was treated with 0.5 ml isopropanol. The mixture was centrifuged at 12,000 g for 10 min at 4°C. The supernatant was discarded, and 0.75 ml 75% ethanol was added, and then centrifuged at 7,500g for 5 min at 4°C. The precipitate containing total RNA was stored at −80°C until further processing. RNA concentration was measured spectrophotometrically.

### Reverse transcription

Total RNA was transcribed to complementary DNA(cDNA) by reverse transcription (RT) with hexamer random primers (Promega, U.S.A). 1 μg of total RNA was added to 0.5 μg of primer. A reaction mixture containing MMLV reverse transcriptase (Promega), buffer solution (Promega), dNTP (Genda, Canada) in a concentration of 0.2 mM/L, and 25U recombinant ribonuclease inhibitor (Huamei, China). The reaction allowed to proceed at 37°C for 60 minutes, and was stopped by heating to 95°C for 5 minutes followed by cooling on ice. The cDNA was stored at −30°C for further procedure.

### Amplification of specific complementary DNA (cDNA)

Specific cDNA products corresponding to mRNA for rat β-actin, MMP-9 were amplified using the polymerase chain reaction (PCR). 1 μl of cDNA was taken for PCR, which was performed in PCR buffer (Genda), using 0.2 mM/L dNTP (Genda), 1 μM/L of both primers (Genda), and 2.0 U Taq DNA polymerase (Genda). GeneAmp2700 Thermal Cycler (U.S.A) was used for amplification with the following sequence profile: initial denaturation at 95°C for 5 minutes followed by relevant number of cycles of three temperature PCR (denaturing, 94°C for 30 seconds; annealing, 65°C for 30 seconds; and extension, 72°C for 30 seconds) ending with a final extension at 72°C for 7 minutes and cooling to 4°C.

The primers sequence for

β-actin:

up 5′-ATGGTGGGTATGGGTCAGAAGG-3′ (located at exon 2),

down 5′-GTACATGGCTGGGGTGTTGAAGG-3′ (located at exon 4).

The length of the amplified segments was 270 bp;

MMP-9 :

up 5′-ATCGACTCCAGTAGACAATCC-3′ (located at exon 9)

down 5′-CAGAGAACTCGTTATCCAAGCG-3′ (located at exon 12)

The length of the amplified segments was 443 bp.

### Gel electrophoresis

The amplified PCR product was identified by electrophoresis of 10 μl sample on 1.5% agarose gel stained with 0.5 μg/ml of ethidium bromide. Sample products were visualized by UV transillumination and the gel was photographed. Specific products were identified by size in relation to a known DNA MARKER (Takara Bio, Dalian, China) run with each gel. MMP-9 cDNA was semiquantitatively analyzed by densitometric comparison to β-actin (internal control) from the same sample after the positive image was digitized by video for computerized densitometry. The results are given as a ratio of intensity of MMP-9 and β-actin mRNA.

### Statistical analysis

All data were expressed as mean ± SD. Statistic analysis was performed with statistical software (SPSS 13.0, Chicago, IL), using One-Way ANOVA and bivariate correlation analysis. Generally p values under 0.05 are considered significant.

## Results

### Functional changes

24-hour urine protein excretion did not differ between the transplant animals and controls. 24-hour urine protein excretion of each group was given as mean ± SD. There was no significant difference between the transplanted animals and two control groups (Table 1).

**Table 1:** 24-hour urine protein excretion, serum creatinie and creatinine clearence in transplant and control animals

**N**	**24-hour urine protein Excretion (g/d)**	**Serum creatinie (μ mol/L)**	**Creatinine clearence (ml/min/100 g bw)**
Transplant animals 10	0.025±0.028	96.2±36.4	0.180±0.097
controls(LEW) 10	0.035±0.016	74.3± 4.9	0.266±0.079^**a**^
controls(F334) 10	0.040±0.024	68.7±6.2^**a**^	0.297±0.070^**b**^

24-hour urine protein excretion did not differ between the transplant animals and F334 controls or LEW controls. The serum creatinine levels significantly differed between transplant animals and F344 controls, but not between transplant animals and LEW controls. The levels of creatinine clearance in transplant animals were significantly higher than those in either F344 controls or LEW controls (a P < 0.05, vs. allografts. b P < 0.01, vs. allografts).

#### Serum creatinine levels in transplant animals and controls

We observed a tendency towards higher serum creatinine levels in transplant rats. The serum creatinine levels differed between transplant animals and F334 controls, but not between transplant animals and LEW controls (Table 1).

#### Creatinine clearance in transplant and control animals

We observed a tendency towards lower creatinine clearance (ml/min/100 g bw) in transplanted rats. Creatinine clearance in transplant animals was significantly higher than those in either F334 controls or LEW controls (Table 1).

### Histological changes

**Figure 1:**
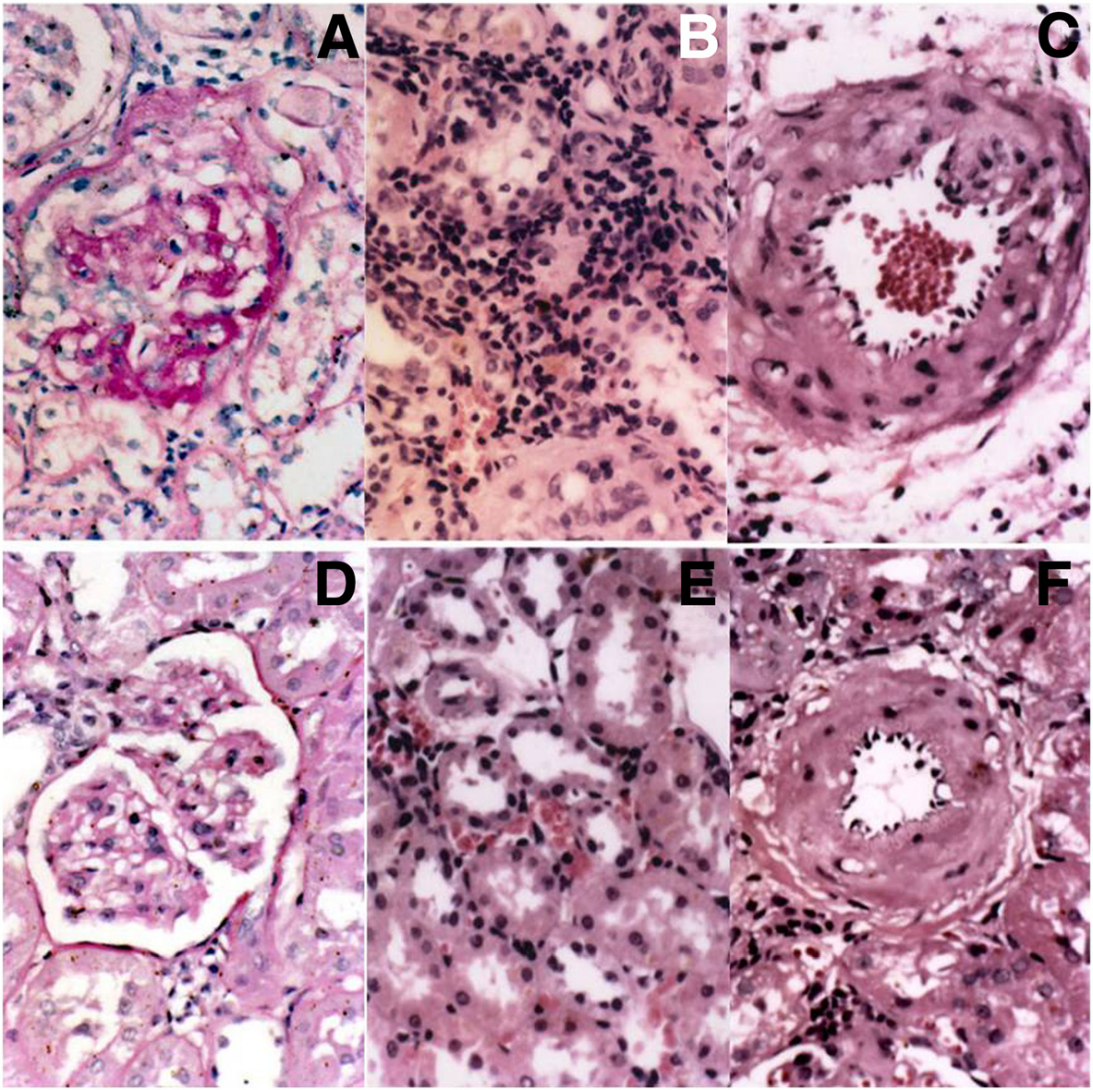
**The renal pathologic change of allografts and controls.** Pathological changes of glomeruli (**A**), tubulointerstitium (**B**), and arteriolar wall (**C**) in allografts and controls (**D**, **E**, and **F**, HE staining × 400).

At week 12 post-transplantation, transplant rats developed a higher degree of mesangial expansion, glomerulosclearosis and adhesions to Bowman’s capsule (Figure 1A), tubulopathy and interstitial mononuclear cells infiltration (Figure 1B), and intimal proliferation in allografts (Figure 1C). No abnormality was observed in glomeruli (Figure 1D), tubulointerstitium (Figure 1E) and the arteries (Figure 1F) of F334 controls and LEW controls.

#### Banff sums in allografts and controls

**Figure 2:**
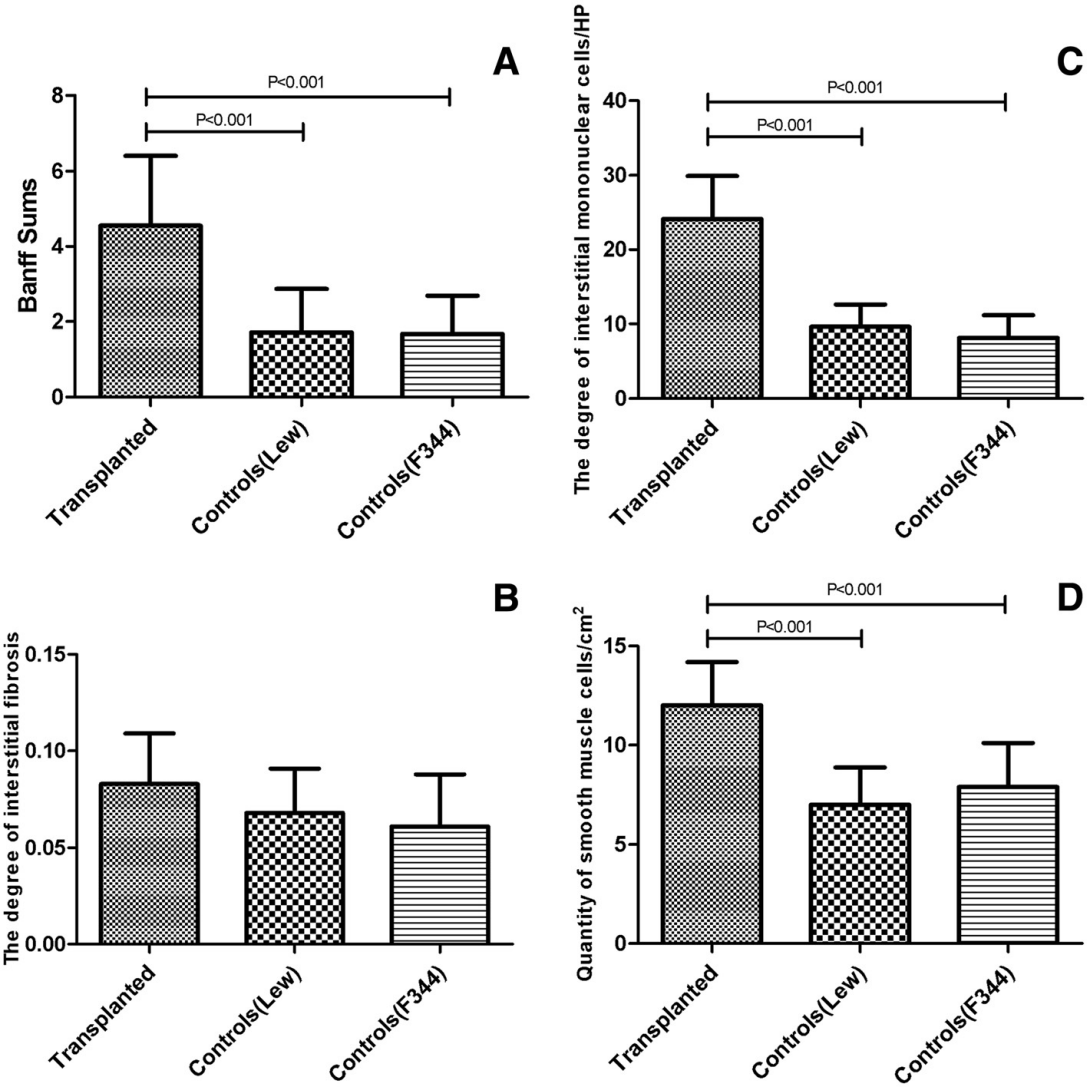
**Histology change in allografts and controls. A** The Banff sums of renal structural damage in allografts and controls. Rats in the transplanted group developed a higher degree of renal interstitial fibrosis, tubulopathy, glomerulopathy and intimal proliferation. The Banff sum of scores in allografts was significantly higher than that in either LEW controls or F344 controls. **B** The percentages of interstitial fibrosis in allografts and controls (100 ×). There was no significant difference between the percentages of interstitial fibrosis of allografts and either F344 controls or LEW controls. **C** The quantity of interstitial mononuclear cells in allografts and controls (400 ×). The quantity of interstitial mononuclear cells in allografts significantly increased compared to that of either F344 controls or LEW controls. **D** The quantity of vascular SMCs in the cortex arteriolar wall of allografs and controls (× 400). The quantity of vascular SMCs in allografts was significantly increased compared to that of either F344 controls or LEW controls.

The sum of these parameters such as glomerulopathy, tubulopathy, interstitial mononuclear cell infiltration, intersitial fibrosis, and intimal proliferation given as Banff sum of scores, was significantly higher in allografts than that in either F334 controls or LEW controls (Figure 2A).

#### Interstitial fibrosis in allografts and controls

There was no significant difference between the percentages of interstitial fibrosis in allografts and either F334 controls or LEW controls (Figure 2B).

#### The quantity of interstitial mononuclear cells in allografts and controls

The quantity of interstitial mononuclear cells in allografts was significantly increased compared to that of controls (Figure 2C).

#### The quantity of vascular smooth muscle cells in the wall of renal cortex arteriole in allografts and controls

The quantity of vascular smooth muscle cells of allografts significantly increased compared to that of the F334 controls and LEW controls (Figure 2D).

### Molecular biology

**Figure 3:**
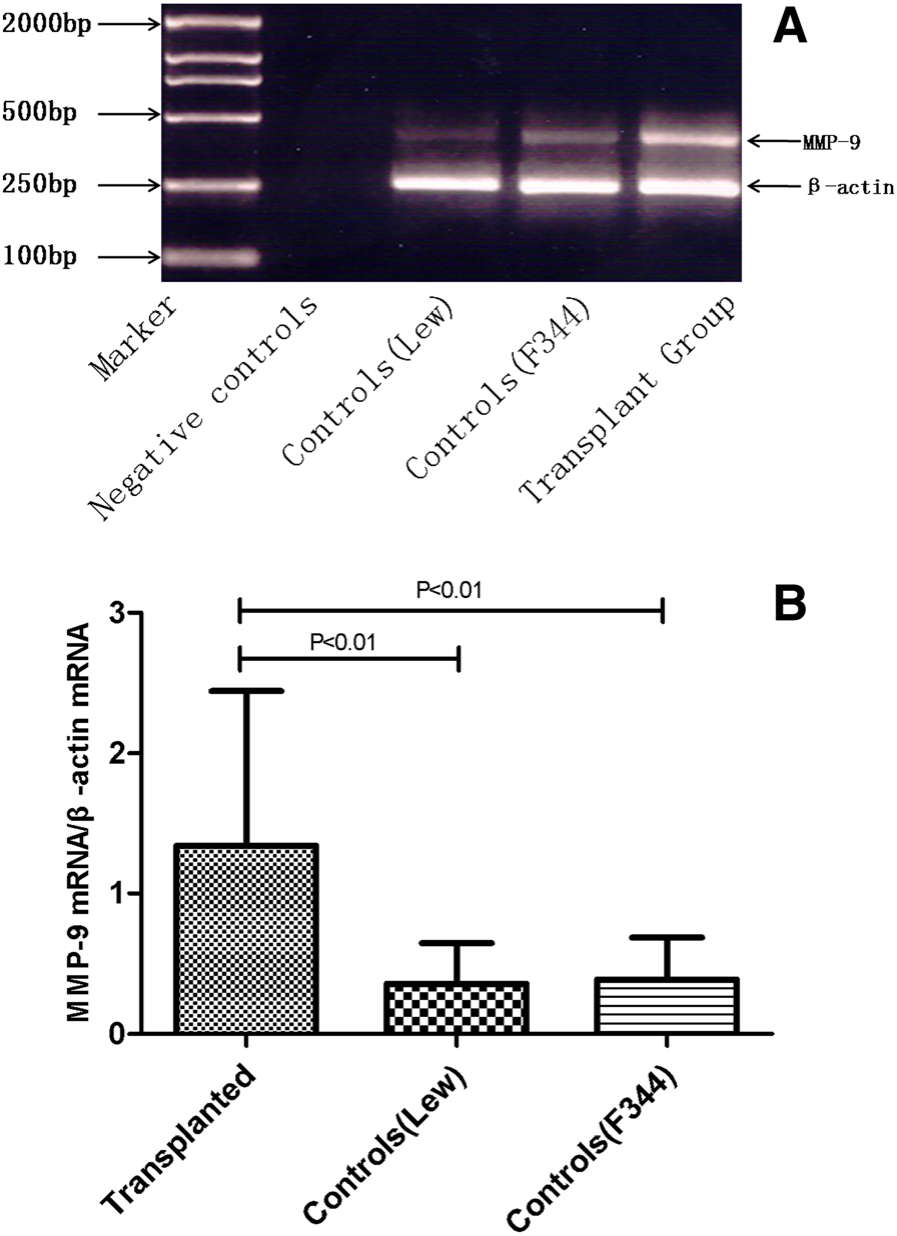
**MMP mRNA expression in allografts and controls.** Analyzed by densitometric comparison to β-actin (internal control) from the same sample after the positive image was digitized by video for computerized densitometry. The results were given as a ratio of the intensity of MMP-9 mRNA and β-actin mRNA. The expression MMP-9 mRNA was significantly increased in allografs compared to either F344 controls (P < 0.05) or LEW controls (P < 0.05).

We observed a tendency towards higher level of MMP-9 mRNA expression in transplant rats. The expression of MMP-9 mRNA significantly increased in allografs compared to either group of controls (Figure 3).

### Immunohistochemistry

**Figure 4:**
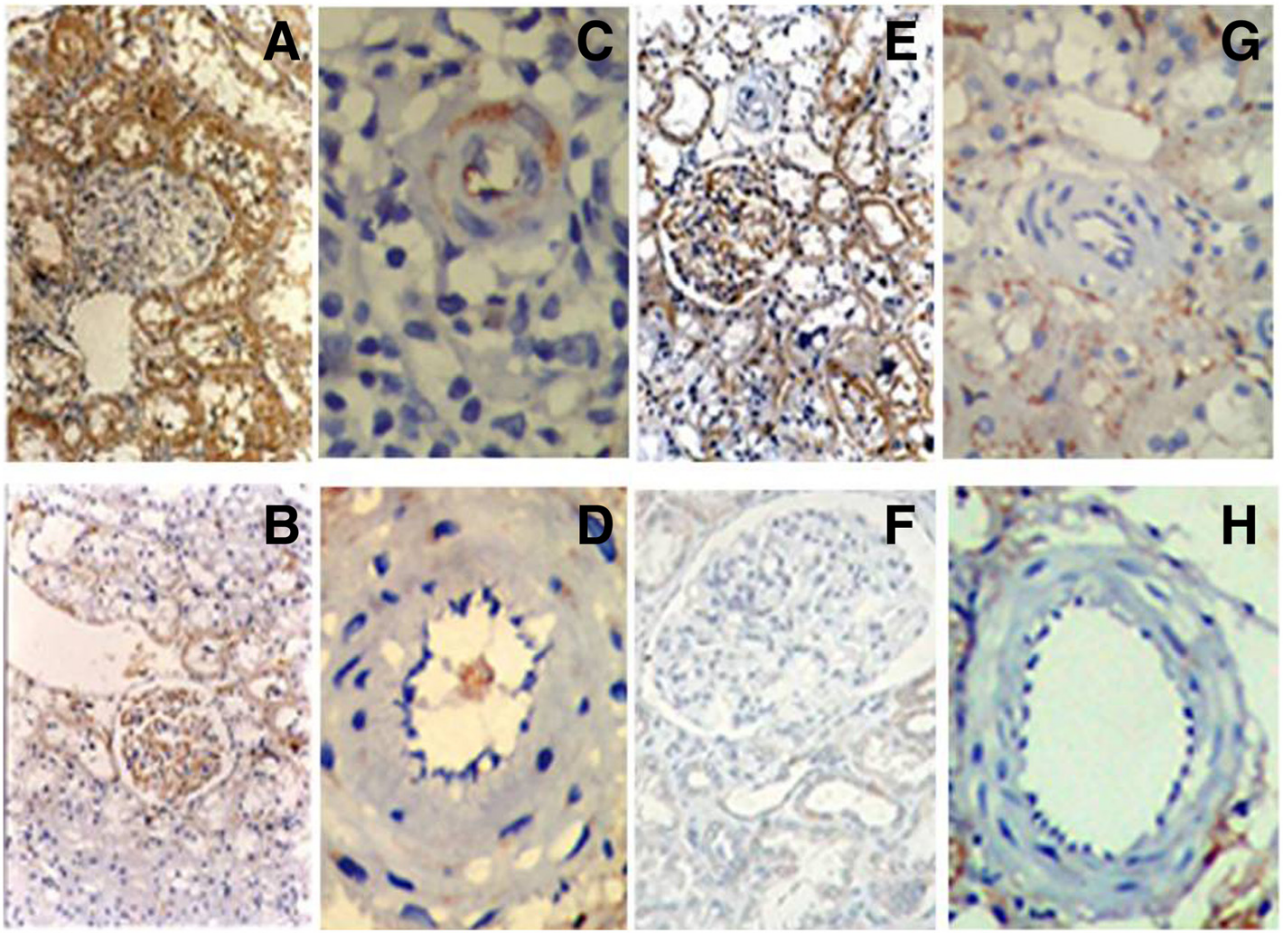
**Immunohistochemical localization of MMP-9 and TGF-beta1 in the kidney of allografts and controls.** Immunohistochemical analysis showed that MMP-9 and TGF-beta1 stained in tubulointerstitium and arteriolar walls of allografts and controls (**A** and **C** are MMP-9 staining in allografts, **B** and **D** are controls. **E** and **G** are TGF-beta1 staining in allografts, **F** and **H** are controls).

MMP-9 and TGF-beta1 immunostaining were observed in glomeruli, tubulaointerstitium, and arterial wall (Figure 4A and 4C are MMP-9 staining in allografts; Figure 4B and 4D are controls. Figure 4E and 4G are TGF-beta1 staning in allografts; Figure 4F and 4H are controls).

### MMP-9 expression in the kidneys of allografts and controls at week 12 post-transplantation

**Figure 5:**
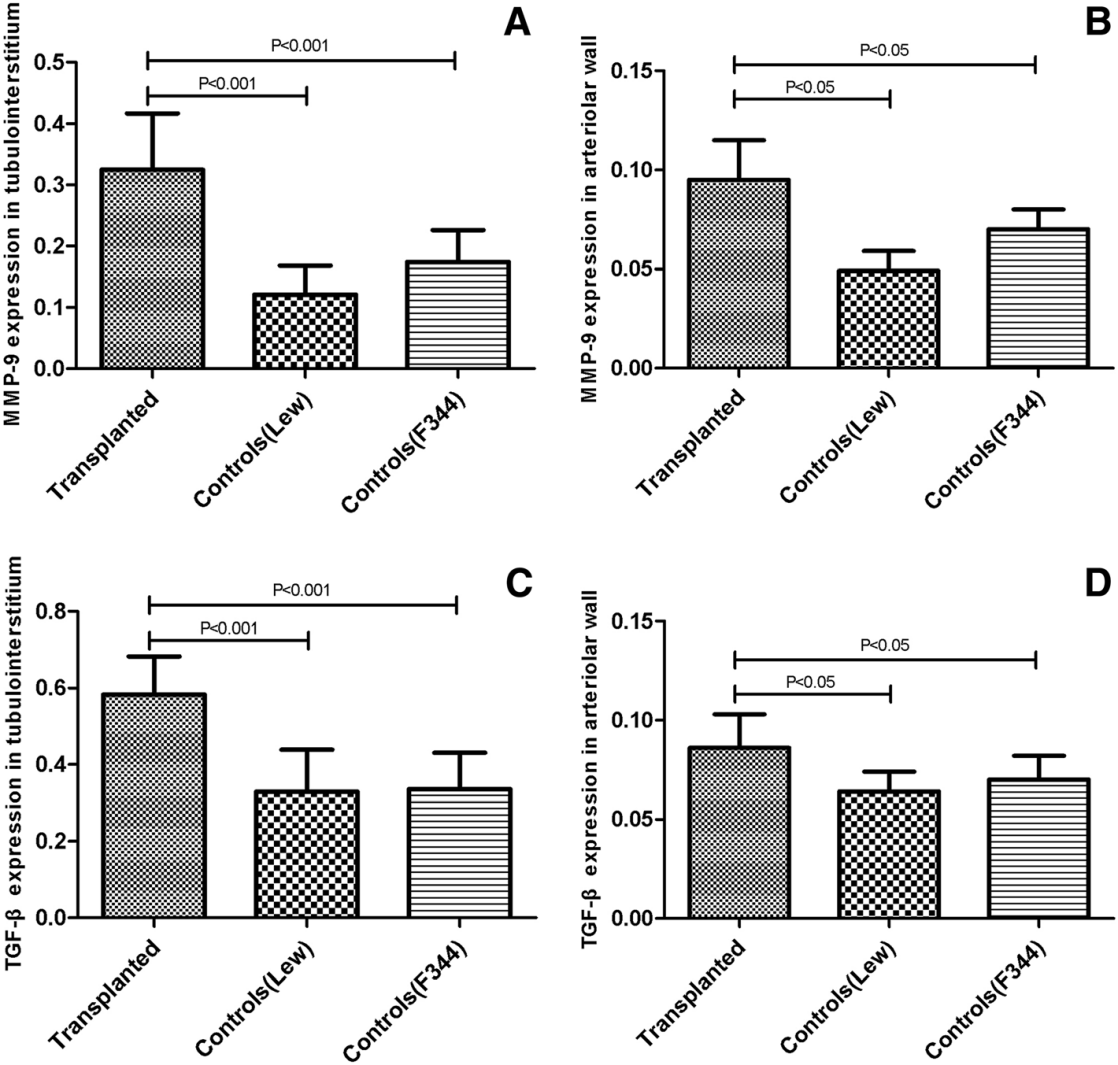
**The expression of MMP-9 and TGF-beta1 in allografts and controls. A** Expression of MMP-9 in the tubulointerstium of allografts and controls (100 ×). . The immunostaining of MMP-9 in tubulointerstitium of allografts (0.325 ± 0.093) significantly increased compared to either F344 controls (0.174 ± 0.052) and LEW controls (0.121 ± 0.047). **B** Expression of MMP-9 in the arteriolar wall of allografts and controls (100 ×). The immunostaining of MMP-9 in arteriolar wall of allografts significantly increased compared to F344 control. **C** Expression of TGF-beta1 in the tubulointerstium of allografts and controls (100 ×).The immunostaining of TGF-beta1 in tubulointerstitium of allografts significantly increased compared to either F344 controls or LEW controls. **D** Expression of TGF-beta1 in the arteriolar wall of allografts and controls (100 ×). The immunostaining of TGF-beta1 in arteriolar wall of allografts significantly increased compared to either F344 controls or LEW controls.

The immunostaining of MMP-9 in tubulointerstitium of allografts (0.325 ± 0.092) was significantly higher than that of F344 controls (0.174 ± 0.052, P < 0.01) and LEW controls (0.121 ± 0.047, P < 0.01) (Figure 5A).

The immunostaining of MMP-9 in arterial wall of allografts (0.095 ± 0.020) was significantly intense, compared to that of F344 controls (0.070 ± 0.010, P < 0.05) and LEW controls (0.049 ± 0.010, P < 0.05) (Figure 5B).

### TGF-beta1 expression in the allografts and controls at 24 weeks

The immunostaining of TGF-beta1 in tubulointerstitium of allografts (0.583 ± 0.100) was significantly intense, compared to that of F334 controls (0.329 ± 0.107, P <0.01) and LEW controls (0.336 ± 0.095, P < 0.01) (Figure 5C).

The immunostaining of TGF-beta1 in arteriolar wall of allografts (0.086 ± 0.017) was significantly intense than that of F344 controls (0.064 ± 0.010, P < 0.05) and LEW controls (0.070 ± 0.012, P < 0.05) (Figure 5D).

### Correlation analysis

#### The expression of MMP-9 mRNA and the degree of mononuclear cells infiltration in allografs

**Figure 6:**
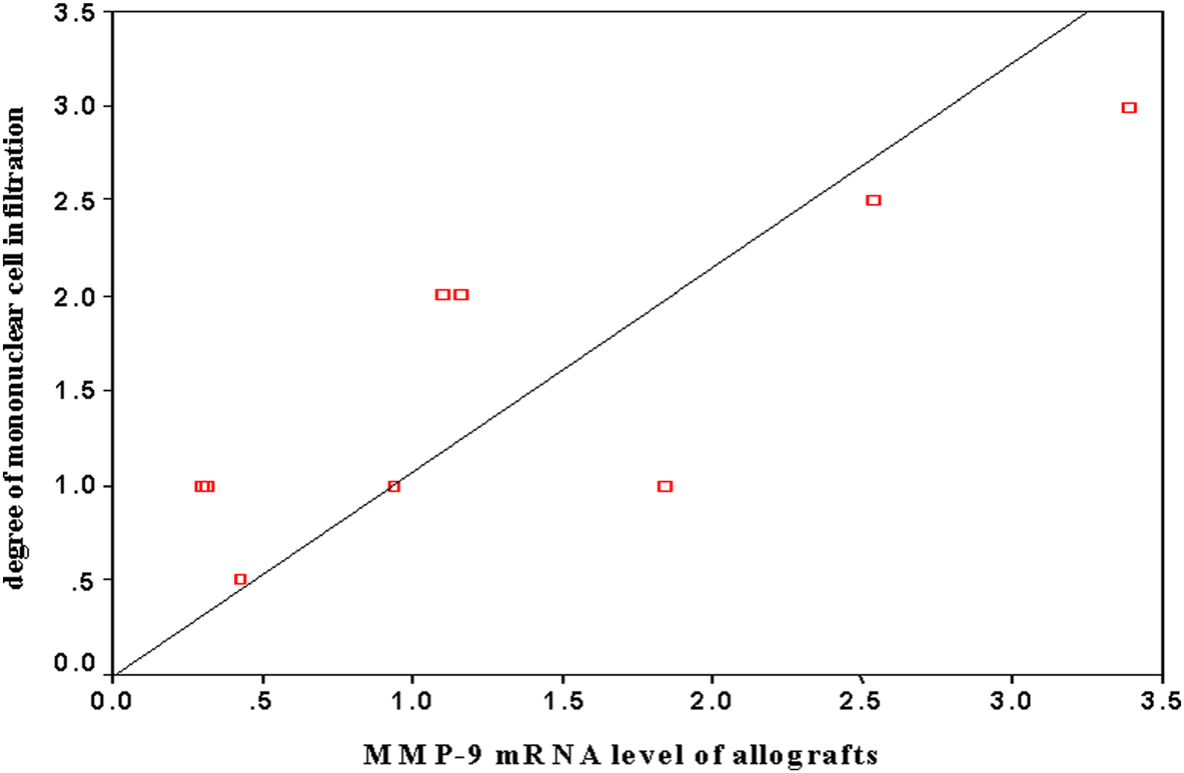
**The correlation between the expression of MMP-9 mRNA and degree of mononuclear cells infiltration in allografts.** MMP-9 mRNA level of allografts was demonstrated by MMP-9 mRNA/β-actin mRNA ratio, the degree of interstitial mononuclear cell infiltration was evaluated according to Banff Criteria, as follow, 0 = No or trivial interstitial inflammation; 1 = up to 25% of parenchyma inflamed; 2 = 26% to 50% parenchyma inflamed; 3 = > 50% parenchyma inflamed. The result of bivariate non-parameter correlation analysis demonstrated: the MMP-9 mRNA levels of allografts were correlated to the degrees of interstitial mononuclear cell infiltration. (P < 0.05, Spearman correlation coefficient was 0.653).

We found positive correlation between the expression of MMP-9 mRNA and degree of mononuclear cells infiltration in allografs by bivariate non-parameter correlation analysis (r = 0.65, p < 0.05, Figure 6).

#### The immunostaining of MMP-9 in tubulointerstitium and the quantity of interstitial mononuclear cells in allografts

**Figure 7:**
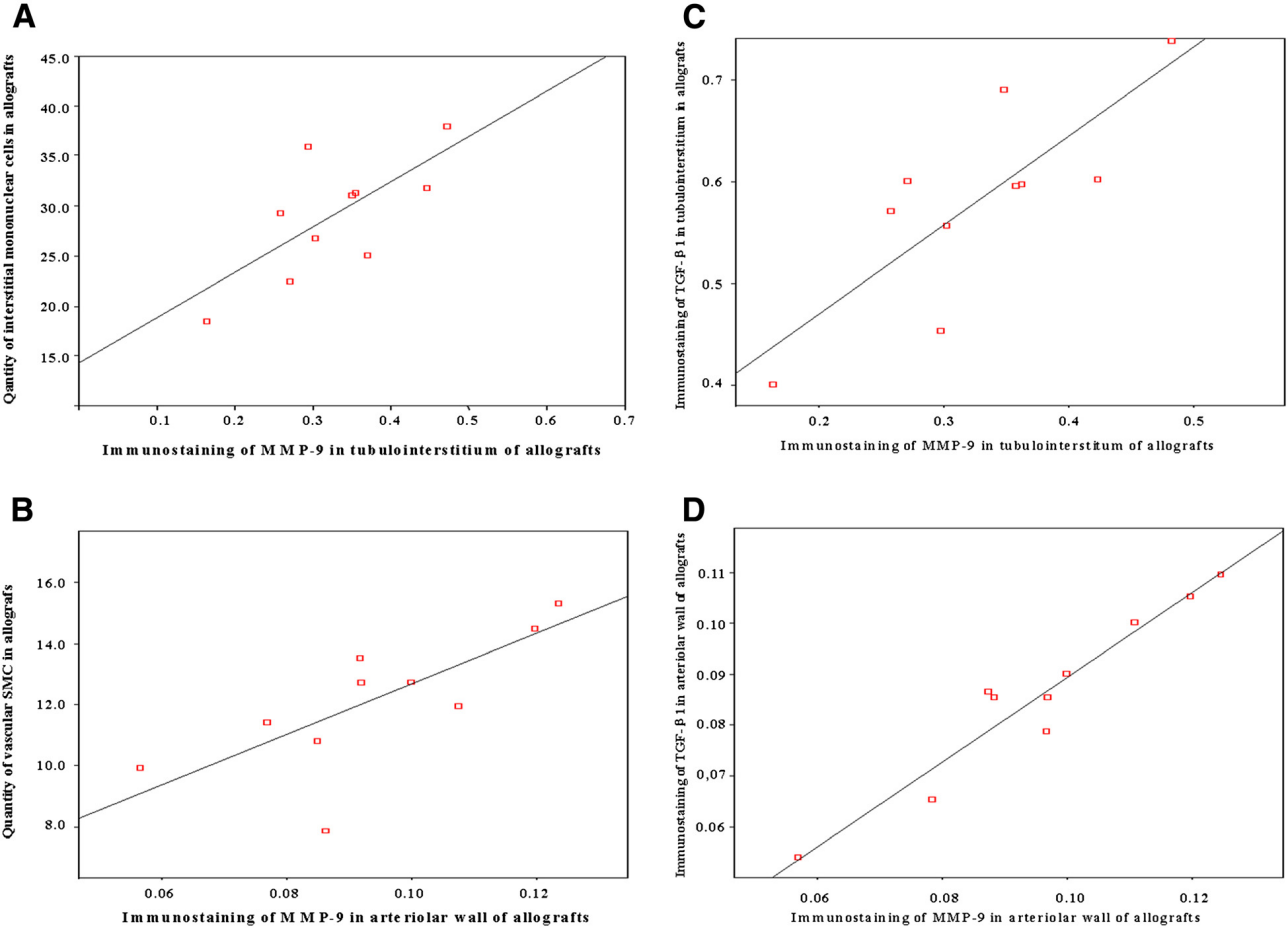
**The potential role of MMP in earlier stage of CAN. A** The correlation between the immunostaining of MMP-9 in tubulointerstitium and the quantity of interstitial mononuclear cells in allografts. The immunostaining of MMP-9 in tubulointerstitium was correlated to the quantity of interstitial mononuclear cells in allografts (P < 0.05, Spearman correlation coefficient was 0.757). **B** The correlation between the immunostaining of MMP-9 in arteriolar wall and the quantity of vascular SMCs in allografts. The immunostaining of MMP-9 in arteriolar wall was correlated to the quantity of vascular SMC in allografts (P < 0.05, Spearman correlation coefficient was 0.741). **C** The correlation between the immunostaining of MMP-9 and TGF-beta1 in the tubulointerstitium of allografts. The immunostaining of MMP-9 and TGF-beta1in tubulointerstitium in allografts have correlation. (P < 0.01, Spearman correlation coefficient was 0.810). **D** The correlation between the immunostaining of MMP-9 and TGF-beta1 in arteriolar wall of allografts. The immunostaining of MMP-9 and TGF-beta1 in arteriolar wall of allografts have correlation. (P < 0.001, Spearman correlation coefficient was 0.958).

We found positive correlation between the immunostaining of MMP-9 in tubulointerstitium and the quantity of interstitial mononuclear cells in allografts by bivariate non-parameter correlation analysis (r = 0.757, P < 0.05, Figure 7A).

#### The immunostaining of MMP-9 in renal arteriolar wall and the quantity of vascular smooth muscle cells in allografts

We found positive correlation between the immunostaining of MMP-9 in arteriolar wall and the quantity of vascular SMCs in allografts by bivariate non-parameter correlation analysis (r = 0.741, P < 0.05, Figure 7B).

#### The immunostaining of MMP-9 and TGF-beta1 in the tubulointerstitium of allografts

We found positive correlation between the immunostaining of MMP-9 and TGF-beta1 in tubulointerstitium of allografts by bivariate non-parameter correlation analysis (r = 0.810, P < 0.01, Figure 7C).

#### The immunostaining of MMP-9 and TGF-beta1 in the arteriolar wall of allografts

We found positive correlation between the immunostaining of MMP-9 and of TGF-beta1 in the arteriolar wall of allografts by bivariate non-parameter correlation analysis (r = 0.958, P < 0.001, Figure 7D).

## Discussion

The etiology of CAN is multifactorial and involves both immune-dependent and immune-independent factors. Immune-dependent factors include cell-mediated immune responses and antibody-mediated responses to donor antigens. Immune-independent factors include donor characteristics, cold ischemia time, cytomegalovirus infection, hypertension, dyslipidemia, and the use of calcineurin inhibitors (CNI), notably CsA, and more recently Tac. In our present study, we didn’t found T-cell infiltration and C4d sediment, so we focus on the immune-independent injury of CAN. There has Ahmedabad Tolerance Induction Protocol reported for treatment of immune injuries of CAN [18], but in death of reports for non-immune injury of CAN, since the mechanism of which still needed to be fully revealed.

In the present study, we investigated the expression of MMP-9 and its role, with F334 to LEW transplant rat model, in the early stage (12 weeks post-transplantation) of chronic allograft nephropathy which represents the most common cause of late graft failure. We also investigated the correlation between MMP-9 expression and the infiltration of mononuclear cells and SMCs replication in the intima of vascular wall which are the cardinal pathological changes of early stage of CAN [9,19,20].

It was shown in our data that MMP-9 expression was upregulated in the early stage of CAN, at the same time there was significant mononuclear cells infiltration in the interstitium of allografts, and the MMP-9 expression was correlated positively to the mononuclear cell infiltration and SMC replication. Studies in other disorders such as tumor, atherosclerosis and many inflammatory diseases indicate that extracellular matrix degradation should be prerequisite for the migration of mononuclear cells and other cells in the tissue, and the ability of MMP-9 to degrade components of the extracellular matrix and to regulate the activity of a number of soluble proteins confers an important role in various physiological and pathological processes [16]. Therefore, the upregulated MMP-9 in the early stage of CAN, showed in our data, might degrade extracellular matrix, which further aggravate the mononuclear cells infiltration, and lead to tubulointerstitial fibrosis and chronic renal failure eventually. We could conclude that MMP-9 might play an important role in the early stage of CAN, the degradation of extracellular matrix caused by increased expression of MMP-9 might be essential for the migration and proliferation of mononuclear cells in the early stage of CAN.

Our data suggest that upregulated MMP-9 expression in the early stage of CAN has significant correlation with SMC replication in the vascular intima of the allografts. Bendeck MP et al. found that administration of a metalloproteinase inhibitor after injury resulted in a 97% reduction in the number of SMCs migrating into the intima in rat carotid artery [21]. Recent research also provides evidence that MMP-9-deficient aortic SMCs had not only decreased the migratory activity, but also decreased the capacity to make collagen contraction compared with wild-type cells [22]. Cho A et al. found a denuding injury to the arteries of wild-type mice promoted the medial SMC replication and the migration of medial SMCs into the neointima, but SMC replication was significantly lower, and SMC migration and arterial lesion growth were significantly impaired in MMP-9 knockout arteries. SMCs, isolated from MMP-9 knockout mouse arteries, showed an impairment of migration and replication in vitro [23]. Thus, based on above evidence, we can reach a conclusion that upregulated MMP-9 expression in the early stage of CAN, shown with our data, may breakdown vascular basement membrane and, therefore, is critical for the development of arterial lesions of CAN by regulating both SMC migration and proliferation.

Our data showed MMP-9 expression was positively correlated to the increased TGF-beta1 expression in the tubulointerstitium and arteriolar wall of the allografts. It is well known that TGF-beta1 is a key fibrogenetic cytokine involved in the pathogenesis of chronic renal allograft dysfunction. It was demonstrated in mammary cancer cells derived from Tenascin-C (TN-C) - deficient mice, that TGF-beta1 induce MMP-9 expression in a dose-dependent manner, and this inducement was significantly enhanced by addition of TN-C. Neutralization with specific anti-TGF-beta1 antibody showed decreased expression of MMP-9, indicating that TGF-beta controls the baseline MMP-9 expression by a direct autocrine mechanism [24]. Kobayashi T et al. found MMP-9 expression by fibroblasts was induced by the addition of TGF-beta1 or tumour necrosis factor (TNF)-alpha to the culture medium [25]. Palosaari H et al. found TGF-beta1 significantly upregulated MMP-9 mRNA in odontoblasts [26]. All the evidences above suggest that TGF-beta1 may be one of the important causes leading to the upregulation of MMP-9 expression in the early stage of CAN.

The pathological mechanism of CAN is complicated; there are many contributors, such as MMPs and TIMPs regulated extracellular matrix (ECM) synthesis and degradation, introducing inflammation cells infiltration. In our previous study [27], we found that matrix metalloproteinase-2 (MMP-2) and tissue inhibitor of metalloproteinase-1 (TIMP-1) were important cytokines for ECM synthesis and degradation, and the excess accumulation of ECM is the main pathological mechanism of fibrosis in antibody-mediated renal graft rejection. Our present study reveals that TGF-beta1 induced expression of mmp-9 may induce mononuclear cells infiltration in the interstitium of allografts and SMC replication. However, the signaling pathway of MMPs and TIMPs regulate the synthesis and degradation of ECM remains to reveal. We are going to study the role of GSK-3β-β-actin signaling pathway, because our pilot study had found that expression of GSK-3β has positive correlation with either inflammatory cell infiltration or interstitial fibrosis/tubular atrophy in human renal allograft tissue [28]. Further studies are needed.

## Conclusions

MMP-9 may play an important role in the mechanism of pathological changes during the earlier period of chronic allograft nephropathy, and its expression may be induced by TGF-beta1.

## Competing interests

The authors declare that they have no competing interest.

## Authors’ contributions

HZ design the study, DG and YS performed research and wrote the first draft of the manuscript, XL and YD participated in the statistical analyses. All the authors read and approved the final manuscript.
